# Sex-related differences in the short and long-term outcome of internal pallidus stimulation for dystonia

**DOI:** 10.1007/s10072-025-08733-3

**Published:** 2026-01-17

**Authors:** Luigi M. Romito, Roberta Telese, Ahmet Kaymak, Fabiana Colucci, Antonio E. Elia, Sara Rinaldo, Grazia Devigili, Roberto Cilia, Giovanna Zorzi, Alberto Mazzoni, Valentina Leta, Michael Zech, Miryam Carecchio, Barbara Garavaglia, Vincenzo Levi, Nico Golfrè Andreasi, Roberto Eleopra

**Affiliations:** 1 Department of Clinical Neurosciences, Movement Disorders Unit, Fondazione IRCCS Istituto Neurologico Carlo Besta, via Giovanni Celoria 11, Milan, 20128 Italy; 2 Department of Pediatric Neuroscience, Fondazione IRCCS Istituto Neurologico Carlo Besta, Milan, Italy; 3 Unit of Medical Genetics and Neurogenetics, Fondazione IRCCS Istituto Neurologico Carlo Besta, Milan, Italy; 4 Functional Neurosurgery Unit Neurosurgery Department, Fondazione IRCCS Istituto Neurologico Carlo Besta, Milan, Italy; 5 Computational Neuroengineering Laboratory, Scuola Superiore Sant’Anna di Pisa, Pisa, Italy; 6 Institute of Neurogenomics, Helmholtz Zentrum München, Neuherberg, Germany; 7 Institute of Human Genetics, School of Medicine, Technical University of Munich, Munich, Germany; 8 Institute for Advanced Study, Technical University of Munich, Garching, Germany; 9 Department of Neuroscience, University of Padua, Padua, Italy; 10 Department of Health Sciences, University of Milan, Milan, Italy; 11 PhD Program in Neuroscience, University of Milano-Bicocca, Milan, Italy; 12 Department of Neuroscience and Rehabilitation, University of Ferrara, Ferrara, Italy; 13 Parkinson’s Centre of Excellence at King’s College Hospital and King’s College London, London, UK

**Keywords:** DBS, Deep brain stimulation, GPi, Globus pallidum internus, Dystonia, Sex, Female, Male, Burke-Fahn-Marsden dystonia rating scale

## Abstract

**Objectives:**

The importance of sex as a determinant of Globus pallidus internus deep brain stimulation (GPi-DBS) outcome in adults with dystonia remains uncertain. We investigated whether sex is a determinant of the efficacy and safety of GPi-DBS in dystonia.

**Methods:**

In this double-center, retrospective cohort study, we followed patients with idiopathic, inherited, or secondary dystonia for at least 12 months after surgery. Dystonia was assessed using the Burke-Fahn-Marsden Dystonia Rating Scale (BFMDRS). Adverse events were recorded.

**Results:**

Fifty-six consecutive patients (22 M/34F) were studied. GPi-DBS led to comparable improvements in both sexes. Male patients showed a motor improvement of 62.6 ± 42.4% at 1 year and 53.9 ± 20.9% at the last follow-up (8.6 ± 3.3 years), while female patients showed a motor improvement of 60.5%±29.0% at 1 year and 48.6 ± 28.3% at the last follow-up (at 7.1 ± 4.2 years). Improvement in disability and adverse events were comparable between the two groups. At the first reglage, females showed lower electrical energy delivery, which increased significantly during follow-up. The position of the optimal stimulation center did not differ significantly between the two groups.

**Conclusion:**

Our study suggests that the overall motor outcomes and safety profiles were similar across sexes, indicating that factors such as age at implant, precision of lead positioning, and severity of dystonia may be more critical determinants of efficacy than sex itself.

**Supplementary Information:**

The online version contains supplementary material available at 10.1007/s10072-025-08733-3.

## Introduction

Evidence shows that deep-brain stimulation (DBS) is effective and safe for patients with dystonia [1, 2]. Patients with isolated dystonia [1] have a remarkable response to globus pallidus internus DBS (GPi-DBS), making it a valuable therapeutic option [2]. Specific analyses of the etiologic, demographic, and phenotypic characteristics that correlate with the efficacy and safety of surgery for childhood- or adult-onset dystonia are clearer for isolated generalized dystonia [2–4]. Shorter symptom duration, lower baseline severity score, and positive *TOR1A* status were all independently associated with significantly better surgical outcomes [2, 4]. Predicting responses to other forms of hereditary, heredodegenerative, or secondary dystonia is less clear [3, 5].

Several works have explored phenotypic presentation, clinical severity, and heterogeneity, as well as treatment response between men and women with dystonia. Overall evidence suggests that, despite differences in clinical phenotypes and the observation that motor symptoms in men with isolated dystonia tend to develop earlier and more severely than in women, the response to oral treatment or botulinum toxin injections leads to comparable improvement between the two sexes [6]. A few studies suggest potential modifications in dystonia symptoms during the menstrual cycle [7] and during pregnancy [6], while menopause and hormone-replacement therapies do not seem to influence dystonic symptoms [7].

The sex-related outcomes of DBS for dystonia are, however, poorly defined. To the best of our knowledge, this topic hasn’t been specifically addressed in any available clinical trial, despite the emerging importance of this biological variable in movement disorder studies [6, 8].

The objective of this study was to evaluate the role of sex in the efficacy and safety of GPi-DBS for dystonia and to identify potential sex differences in the progression of dystonic symptoms during continuous DBS.

## Materials and methods

### Study design and participants

This two-center (Carlo Besta Institute-INCB in Milan and S. Maria della Misericordia University Hospital in Udine, Italy), retrospective, cohort study included adult patients (≥ 16 years of age at surgery) with a clinical diagnosis of dystonic syndrome [1] who were followed for at least 12 months after bilateral GPi-DBS. Patients with secondary etiologies (i.e., dyskinetic-dystonic cerebral palsy, DCP) were excluded if they had marked spasticity (modified Ashworth score ≥ 3 in more than two body segments) [5]. Patients with cognitive or psychiatric disorders or extensive brain MRI damage were also excluded. However, slight abnormalities on T1-weighted images were accepted in patients with DCP.

All patients were tested for mutations in dystonia genes using a customized next-generation sequencing (NGS) panel or, in selected cases, whole exome sequencing [4]. The customized gene panel for dystonia in use at INCB includes the following genes: TOR1A, THAP1, PRKRA, TAF1, TIMM8A, GCH1, TH, SGCE, ATP1A3, PNKD, PRRT2, SLC2A1, CIZ1, ANO3, GNAL, NKX2-1, ATM, HPCA, COL6A3, KCTD17, ADCY5, GNAO1, PNKP, CACNA1A, GRIN1, AOPEP, KMT2B, GLB1, TUBB4A, VPS16, SCP2, PDE10A.

### Clinical evaluation of outcomes

Dystonia was assessed using the motor and disability sections of the Burke-Fahn-Marsden Dystonia Rating Scale (BFMDRS-M and BFMDRS-D) [9]. Patients were evaluated only during stimulation to prevent clinical deterioration. A patient was considered to have improved after DBS if he or she showed at least a 25% reduction in preoperative dystonia severity at 12 months FU [4].

Stimulation settings were recorded 1–3 months after implant and then yearly. Adverse events were collected via patient and caregiver interviews.

### Statistical analysis

Demographic and clinical characteristics were compared between the male and female groups. The Shapiro‒Wilk test was used to assess the normality of numerical variables, with the expectation of a normal distribution in both groups for each variable. Numerical variables with a normal distribution are indicated as the mean ± standard deviation, while others are represented as median ± interquartile range values. Subsequently, the Bartlett test was performed on normally distributed variables to evaluate the comparability of variances between the groups. For the numerical variables in which both groups presented normal distribution and equal variances, unpaired or paired Student’s t-tests were used. For the remaining variables, the Mann‒Whitney U test and Wilcoxon signed-rank test were utilized for unpaired and paired nonparametric comparisons, respectively. Paired comparisons were conducted between baseline and postoperative motor/disability scores at each follow-up. Categorical variables with more than three categories were initially assessed using the chi-square test (χ2). Subsequently, Fisher’s exact test was performed for each category in a post hoc manner.

Spearman’s correlation coefficient was measured to evaluate the linear relationship between variables. To address the statistical significance of the degree of correlation, we conducted a permutation test. Statistical significance was determined with α = 0.05, and all p values were corrected with Holm‒Bonferroni correction.

### Surgical procedures

The DBS electrodes were implanted according to standard procedures in the ventral-posterolateral portion of the GPi. DBS procedure’s technical details have been published before [4, 5].

### Stimulating contact position and optimal stimulation position analysis

The location of the therapeutic contacts and the volume of tissue activated by the surrounding electrical field were determined in a consistent subset of patients (15 M/23F) by combining the preoperative and postoperative high-resolution neuroimages and processing them using the Lead DBS v2.5.2 toolbox (https://www.lead-dbs.org/) [10]. We analyzed the position of the stimulation center at the last follow-up (for monopolar stimulation, the active contact; for double monopolar and interleaved stimulation strategies, the median point between these two contacts) and its effects on the BFMDRS-M scores. We used the Distal Atlas [11] in the Montreal Neurological Institute space (*p* > 0.5 thresholds for defining nuclei borders) integrated into the Lead DBS v2.5.2 suite [10] and postoperative brain MRI or CT images. The technical details of the optimal stimulation targets are in eSupplementary Material S1.

## Results

### Population demographic and clinical characteristics

Fifty-six patients (22 males/34 females) were included in this study from the original registry of 83 dystonic patients who underwent DBS-GPi (38 M/45F). Of these, 26 patients had idiopathic dystonia (10 M/16F), 19 had non-degenerative inherited dystonia or heredodegenerative dystonia (7 M/12F), and 11 had dyskinetic dystonic cerebral palsy (5 M/6F). Twenty-seven patients (16 M/11F) were excluded from the study for single or combined reasons: incomplete baseline data (10 M/3F), incomplete/missing follow-up (FU) data (10 M/9F), and/or early (< 1Y since surgery) lead removal due to infection (1 M/2F).

eSupplemental Table 1 shows the demographics, dystonia features and distribution, and genetic status of the included patients. Age at onset, disease duration at implant, and FU duration (mean 7.7 ± 3.9 years, reaching up to 10–14 years in a few cases) were comparable between the sexes.

At baseline, female patients were older and had lower body weight and height (eSupplemental Tables 1 and 2). There were no differences between the sexes in the distribution of dystonia; however, segmental distribution was more common in females (12 vs. 1). No intersex differences were found in the BFMDRS motor or disability subscales at baseline and during FU, or in the etiology of dystonia (eSupplemental Tables 1 and 3).

### Main result, motor and disability outcome, stratified according to the patient’s sex

**Fig. 1:**
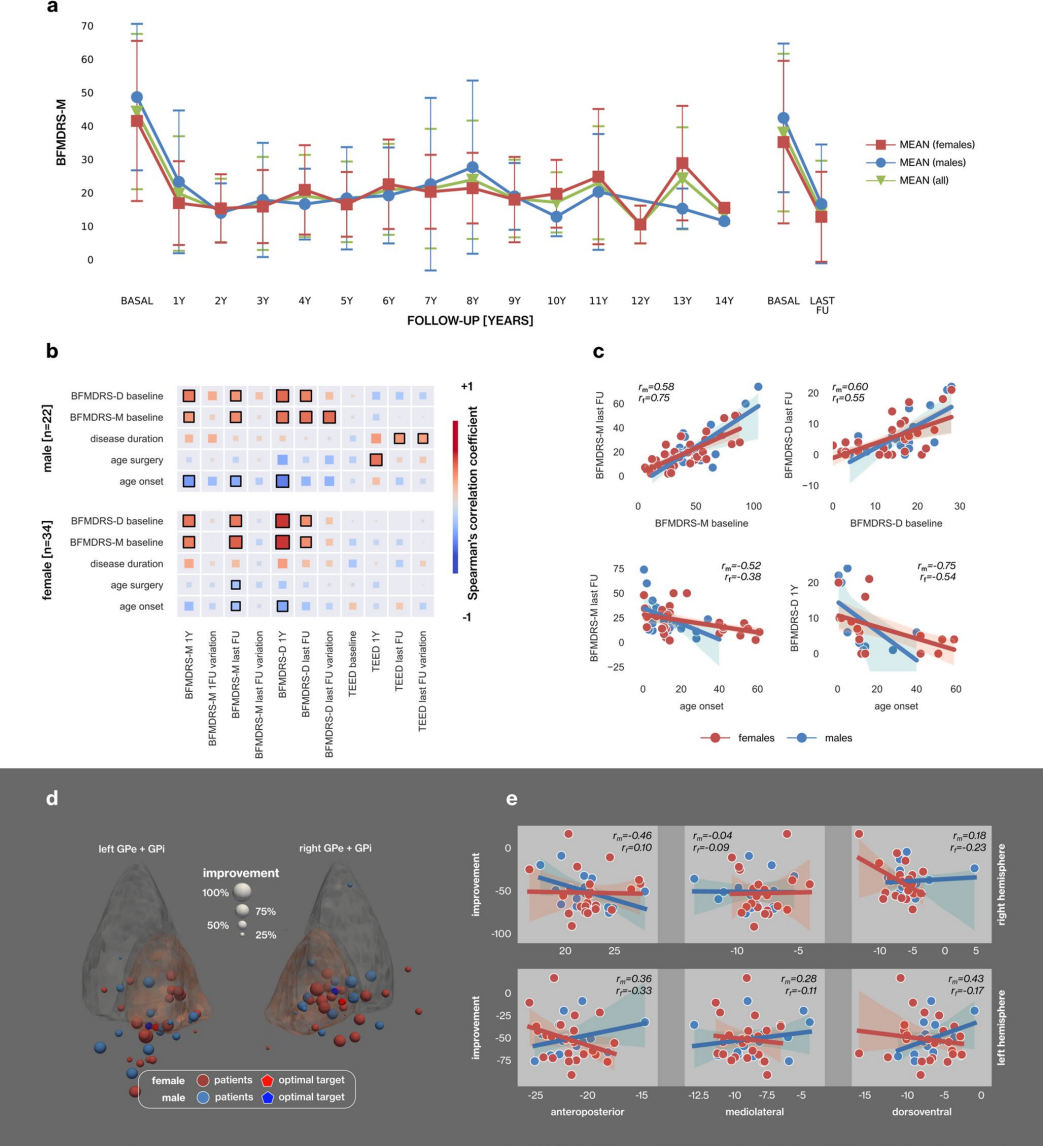
Longitudinal analysis of BFMDRS-M scores, relationship between baseline and follow-up characteristics, and spatial analysis of stimulation centers in dystonia patients. (**a**) The changes observed in BFMDRS-M scores from baseline to the last follow-up assessment (up to 14 years) are depicted with color-coded line plots for female, male, and overall populations. The mean BFMDRS-M scores are represented by squares for females, circles for males, and triangles for all patients, with standard deviations indicated by error bars. (**b**) The correlation matrices illustrate the relationships between baseline and postoperative characteristics in female and male dystonia patients using Spearman’s correlation coefficient. The size and color of the marker indicate the magnitude of the correlation, with a black border indicating significance (*p* ≤ 0.05, permutation test). (**c**) Regression plots display significant correlations between selected clinical and demographic characteristics. Scatter plots are color-coded based on the sex of the patients, with corresponding correlation coefficients indicating significant relationships for male and female patients individually. (**d**) The stimulation center points in the right and left GPi are indicated for male and female patients at the last follow-up evaluation. The size of each point corresponds to the observed improvement in the BFMDRS-M score. The optimal targets, based on weighted centers, are also indicated. (**e**) The linear relationships between the observed improvement and the position of the stimulation center in the anteroposterior, mediolateral, and dorsoventral anatomical axes of the GPi are presented with regression plots. No significant correlation was detected (*p* > 0.05, permutation test)

GPi-DBS resulted in similar motor improvement for both sexes. Male patients showed an improvement of 62.6 ± 42.4% at 1 year and 53.9 ± 20.9% at the last FU (at 8.6 ± 3.3 years). Female patients showed an improvement of 60.5 ± 29.0% at 1 year and by 48.6 ± 28.3% at the last FU (at 7.1 ± 4.2 years) (Table 1; Fig. 1 panel a, eSupplemental Fig. 1).

**Table 1:** Main results. Numerical variables with a normal distribution and comparable variances between the male and female groups were compared with unpaired student’s t-test and the corresponding statistics are presented as the means ± standard deviations. Nonnormal numerical variables were compared with the Mann‒Whitney U test between two groups, and population statistics are indicated as the median ± interquartile range. Categorical variables were tested with fisher’s exact test. Pairwise comparisons were conducted between baseline and follow-up evaluations within patient groups using paired student’s t-test for parametric comparisons and the Wilcoxon signed-rank test for nonparametric comparisons. Significant effects are highlighted

Index	MALE		FEMALE		TOTAL POPULATION		SIGNIFICANCE FOR SEX DIFFERENCES	STATISTICAL TEST
# pts at baseline, 1Y FU and LFU*	22		34		56		--	
BFMDRS-M at baseline	48.6 ± 21.9		41.5 ± 24.0		44.3 ± 23.2		*0.266*	*Student’s t-test*
BFMDRS-M at 1Y FU	14.5 ± 25.6		12.7 ± 15.9		13.5 ± 19.0		*0.577*	*Mann‒Whitney U*
BFMDRS-M at 3Y FU*	11.5 ± 6.8		14.2 ± 15.4		12.0 ± 12.0		*0.884*	*Mann‒Whitney U*
BFMDRS-M at 5Y FU*	12.5 ± 12.9		18.0 ± 16.4		16.0 ± 15.5		*0.907*	*Mann‒Whitney U*
BFMDRS-M at last FU	23.3 ± 17.5		19.5 ± 13.3		21.0 ± 15.0		*0.459*	*Student’s t-test*
BFMDRS-D at baseline	17.3 ± 6.9		13.2 ± 7.8		14.7 ± 7.7		*0.074*	*Student’s t-test*
BFMDRS-D at last FU	6.8 ± 6.4		4.9 ± 5.9		5.7 ± 6.1		*0.267*	*Student’s t-test*
TEED at first reglage (6–8 months)	139.9 ± 94.4		100.2 ± 69.8		118.1 ± 86.0		***0.043***	*Mann‒Whitney U*
TEED at last FU	214.3 ± 314.55		189.7 ± 155.6		206.0 ± 185.5		*0.795*	*Mann‒Whitney U*
Botulinum Toxin treatment, at baseline	8/22		17/34		25/56		*0.412*	*Fisher-Exact test*
Botulinum Toxin treatment, at last FU	3/22		6/34		9/56		*0.459*	*Fisher-Exact test*
Botulinum toxin, % stopped	62.5%		64.7%		69,5%		*1.000*	*Fisher-Exact test*
Adverse events	6/22		5/34		11/56		*0.310*	*Fisher-Exact test*
**Pairwise Nonparametric Comparisons**								
		*p-value*		*p-value*		*p-value*		
BFMDRS-M % variation from baseline to the 1Y FU	−62.6 ± 42.4%	***0.000***	−60.5 ± 29.0%	***0.000***	−61.0 ± 31.3%	***0.000***	*0.967*	*Mann‒Whitney U*
TEED % variation from baseline to the last FU	33.2 ± 404.3%	*0.054*	97.4 ± 241.1%	***0.000***	84.7 ± 315.8%	***0.000***	*0.598*	*Mann‒Whitney U*
**Pairwise Parametric Comparisons**								
		*p-value*		*p-value*		*p-value*		
BFMDRS-D % variation from baseline to the last FU	−61.4 ± 31.8%	***0.000***	−64.0 ± 35.4%	***0.000***	−63.0 ± 33.8%	***0.000***	*0.806*	*Student’s t-test*
BFMDRS-M % variation from baseline to the last FU	−53.9 ± 20.9%	***0.000***	−48.6 ± 28.3%	***0.000***	−50.7 ± 25.6%	***0.000***	*0.459*	*Student’s t-test*

*At 3Y FU, 14 M and 16 F; at 5Y FU, 10 M and 14 F

Female patients showed similar improvement in disability and comparable discontinuation rates of botulinum toxin after implantation compared to males (Table 1).

Linear relationships between baseline demographic and clinical characteristics and changes in stimulation during FU were investigated. Baseline BFMDRS-M scores were found to be significantly correlated with BFMDRS-M scores measured at the last follow-up visit for both groups (r coefficients: rmale = 0.58, rfemale = 0.75, p ≤ permutation test), as well as with BFMDRS-D scores (Fig. 1 panels b and c). The change in BFMDRS-D score at the last follow-up (FU) evaluation compared to the baseline evaluation was associated with the baseline BFMDRS-M score in male patients (rmale = 0.7), but not in female patients (Fig. 1 panel b). Older age at onset was linked to lower BFMDRS-M scores at the last follow-up in both groups (rmale=−0.52, rfemale=−0.38, p ≤ permutation test) (Fig. 1 panel c). Older age at surgery and longer disease duration were linked to greater TEED at last FU, and greater changes in TEED were observed from baseline to the last FU. This was significant only in male patients (Fig. 1 panel b).

The total electrical energy delivered (TEED) at the first *reglage* was lower in female patients; however, the TEED at the last FU was comparable between the two groups (Table 1, eSupplemental Tables 2 and 3).

The position of the optimal stimulation center did not differ significantly between the two groups (Fig. 1 panel d). Our findings did not indicate a strong effect of the spatial position of stimulation on improvement for male and female patients (p ≤ permutation test) (Fig. 1 panel e).

No serious AEs were observed in either sex, and the difference was not statistically significant (males vs. females, 27.3% vs. 14.7%, respectively, *p* = 0.310).

## Discussion

This is the first study to investigate the role of sex in outcomes and feature evolution after GPi-DBS in adult dystonic patients.

Our study found that more females than males underwent GPi-DBS, regardless of age at onset. This aligns with literature data showing that more females are globally affected by dystonia [12] and that the older the age at onset, the greater the proportion of females affected compared to males [13].

The motor outcome was comparable between male and female patients, and the core improvement was maintained up to 14 years after implantation, regardless of sex. A few patients experienced a decrease in motor benefit after more than three years. This phenomenon was not correlated with technical or device problems, and it’s possible that this subgroup of patients was affected by more severely progressive dystonia. This observation aligns with previous studies on the long-term outcome of GPi-DBS in adult or childhood primary or inherited dystonia [2–4] and acquired dystonia patients [5, 14].

In male dystonia patients, age at surgery showed a positive correlation with TEED at the first-year evaluation; in contrast, dystonia duration was significantly positively correlated with TEED at the last follow-up and with the percentage change in TEED from baseline to the final visit (Fig. 1 panel b). However, we don’t know any clinical explanation for this. As reported in Table 1, the percentage change in TEED from baseline to the last follow-up is significant in female patients (97.4 ± 241.1%, *p* ≤ 0.000), whereas in male patients the change is borderline non-significant (33.2 ± 404.3%, *p* = 0.054) after multiple comparison correction. However, these represent within-group differences (females and male patients separately). When comparing the percentage TEED variation between male and female patients, no significant difference was observed (*p* = 0.598, Mann–Whitney Test). Future studies evaluating a wider population may reveal significant sex differences in TEED, which could highlight potential sex-specific mechanisms in circuit plasticity after DBS, as well as in the preoperative and postoperative functional connectome/dysfunctome [15, 16].

All patients underwent scheduled evaluations and assessments, allowing for group comparisons. We analyzed the short- and long-term effects of GPi-DBS in men and women, assessing changes over time. Both hardware- and stimulation-related adverse events and side effects were reported, but the safety outlook was reassuring and similar between sexes, even in long-term FU.

However, our study has several limitations: several FUs are missing and scattered over time, and a large group of patients lacks follow-up data for 5–8 years, which introduces the potential for bias in the interpretation of long-term results. However, the lack of FU data was similar between men and women, suggesting that this bias may have had little effect on our results. Our sample size was relatively small, limiting the statistical analyses between sexes. Age groups (at onset, at implant) were heterogeneous. Another limitation is the use of the BFMDRS to assess acquired dystonia, although this has been done in several studies [5, 14].

Our study suggests that the overall motor outcomes and safety profiles were similar across sexes, indicating that factors such as age at implant, precision of lead positioning, and severity of dystonia may be more critical determinants of efficacy than sex itself, thus highlighting the need for further research to explore the interactions between sex and other factors in dystonia treatment personalization [6].

## Supplementary Information

Below is the link to the electronic supplementary material.

Supplementary Material 1 (DOCX 49.8 KB)

## Data Availability

The data that support the findings of this study are available from the corresponding author upon reasonable request.

## Declarations

### Generative AI and AI-assisted technologies in the writing process’

The authors did not use any generative AI or AI-assisted technology in the writing process during the preparation of this paper.
