# Supplementary material for: Sex-related differences in the short and long-term outcome of internal pallidus stimulation for dystonia
Source: Neurol Sci. 2026 Jan 17;47(1):168. doi: 10.1007/s10072-025-08733-3 (PMC12811284; doi:10.1007/s10072-025-08733-3)
Supplement: Supplementary file 1 — Supplementary Material 1 (DOCX 49.8 KB) [file 10072_2025_8733_MOESM1_ESM.docx]

*eSupplemental Tables, eFigures, and eMaterials*

**Sex-Related Differences in the Short and Long-Term Outcome of Internal Pallidus Stimulation for Dystonia**

**eSupplemental Tables, eFigures, and eMaterials**

**eSupplemental Table 1.** Study population, demographic, and clinical characteristics.

**eSupplemental Table 2.** Statistical comparison of clinical, demographic, and stimulation variables between male and female dystonia patients.

**eSupplemental Table 3.** Pairwise comparison of clinical and stimulation variables from baseline to the 1-year and last follow-up evaluations between male and female dystonia patients.

**eSupplemental Figure 1.** Individual BFMDRS-M scores at baseline and FUs

**eSupplemental Material S1.** Optimal stimulation targets analysis

**eSupplemental Table 1. Study population, demographic, and clinical characteristics**. Numerical variables with a normal distribution and comparable variances between the male and female groups were compared with unpaired Student’s t-test and the corresponding statistics are presented as the mean±standard deviations. Nonnormal numerical variables were compared between two groups with the Mann‒Whitney U test, and population statistics are presented as the median±interquartile range. Categorical variables were tested with chi-square (χ2) and Fisher's exact tests. Significant effects are highlighted.

| Index | MALES | FEMALES | TOTAL POPULATION | SIGNIFICANCE FOR SEX DIFFERENCES | STATISTICAL TEST |
| --- | --- | --- | --- | --- | --- |
| **# pts** | 22 | 34 | 56 | -- |  |
| **Age at onset (Y)** | 13.0±14.0 | 14.0±31.25 | 14.0±27.0 | *0.084* | *Mann‒Whitney U* |
| **Age at surgery (Y)** | 35.0±13.6 | 45.8±13.0 | 45.8±13.0 | ***0.004*** | *Student’s t-test* |
| **Disease duration at implant (Y)** | 18.0±16.0 | 17.5±17.75 | 18.0±16.0 | *0.530* | *Mann‒Whitney U* |
| **FU duration (Y)** | 8.6±3.3 | 7.1±4.2 | 7.7±3.9 | *0.146* | *Student’s t-test* |
| **Weight at baseline (kgs)** | 68.2±10.9 | 54.6±10.7 | 60.4±12.6 | ***0.000*** | *Student’s t-test* |
| **Height at baseline (cms)** | 171.6±7.4 | 160.7±8.7 | 165.4±9.8 | ***0.000*** | *Student’s t-test* |
| **BFMDRS-M at baseline** | 48.6±21.9 | 41.5±24.0 | 44.3±23.2 | *0.266* | *Student’s t-test* |
| **BFMDRS-D at baseline** | 17.3±6.9 | 13.2±7.8 | 14.7±7.7 | *0.074* | *Student’s t-test* |
| **Dystonia Distribution** |  |  |  | ***0.037*** | *Chi-square test* |
| **Generalized** | 16 | 18 | 34 | *0.169* | *Fisher-Exact test* |
| **Segmental** | 1 | 12 | 13 | ***0.009*** | *Fisher-Exact test* |
| **Cervical** | 2 | 3 | 4 | *1.000* | *Fisher-Exact test* |
| **Multisegmental** | 3 | 1 | 4 | *0.289* | *Fisher-Exact test* |
| **Etiology** |  |  |  | *0.891* | *Chi-square test* |
| **Idiopathic (unknown cause)** | 10 | 16 | 26 | *1.000* | *Fisher-Exact test* |
| Sporadic | 9 | 14 | 23 | -- |  |
| Familial | 1 | 2 | 3 | -- |  |
| **Inherited/Heredodegenerative** | 7 | 12 | 19 | *1.000* | *Fisher-Exact test* |
| *AOPEP* | 1 | 0 | 1 |  |  |
| *GNAL* | 0 | 1 | 1 | -- |  |
| *KMT2B* | 1 | 2 | 3 | -- |  |
| *PANK2* | 0 | 1 | 1 | -- |  |
| *SGCE* | 2 | 1 | 3 | -- |  |
| *THAP1* | 0 | 3 | 3 | -- |  |
| *TOR1A* | 2 | 1 | 3 | -- |  |
| *VPS16* | 1 | 3 | 4 | -- |  |
| **Secondary/acquired** | 5 | 6 | 11 | *0.735* | *Fisher-Exact test* |
| Dyskinetic-dystonic Cerebral Palsy (DCP) | 5 | 6 | 11 | *0.735* | *Fisher-Exact test* |

e**Supplemental Table 2. Statistical comparison of clinical, demographic, and stimulation variables between male and female dystonia patients.** Variables between the two groups were compared with the *Mann‒Whitney U test* with *Holm–Bonferroni* correction, and significant differences were highlighted.

| Index | TOTAL POPULATION | | | ETIOLOGY:  GENETIC/  HETERODEGENARATIVE | | | ETIOLOGY:  IDIOPATHIC | | | ETIOLOGY:  ACQUIRED | | |
| --- | --- | --- | --- | --- | --- | --- | --- | --- | --- | --- | --- | --- |
| **# pts** | 34 | | | 19 | | | 26 | | | 11 | | |
|  | ***male*** | ***female*** | ***p-value*** | ***male*** | ***female*** | ***p-value*** | ***male*** | ***female*** | ***p-value*** | ***male*** | ***female*** | ***p-value*** |
| **Weight at baseline (kgs)** | 67.5 | 50.0 | ***0.000*** | 76 | 57 | ***0.008*** | 64.5 | 50.0 | ***0.024*** | 60.0 | 47.5 | *0.054* |
| **Height at baseline (cms)** | 170.0 | 162.0 | ***0.003*** | 177.5 | 164.0 | ***0.010*** | 170.0 | 159.0 | ***0.010*** | 169.0 | 161.5 | *0.333* |
| **TEED at 1Y FU** | 302.5 | 199.0 | ***0.022*** | 275.7 | 198.5 | *0.142* | 576.8 | 166.6 | ***0.019*** | 275.3 | 185.7 | *0.905* |
| **BFMDRS-M at baseline** | 49.5 | 56.3 | *0.986* | 39.0 | 52.7 | *0.536* | 38.2 | 27.0 | ***0.040*** | 73.0 | 58.5 | *0.429* |
| **BFMDRS-D at baseline** | 18.0 | 17.0 | *0.442* | 17.0 | 18.0 | *0.691* | 15.5 | 6.0 | ***0.038*** | 27.0 | 14.0 | *0.052* |

**eSupplemental Table 3. Pairwise comparison of clinical and stimulation variables from baseline to the 1-year and last follow-up evaluations between male and female dystonia patients.** Variables between the two groups were compared with the Wilcoxon signed-rank test with Holm‒Bonferroni correction and significant effects are highlighted.

| Index | MALES | | FEMALES | | TOTAL POPULATION | |
| --- | --- | --- | --- | --- | --- | --- |
| **BODY DISTRIBUTION: GENERALIZED** | | | | | | |
| **BFMDRS-M variation from baseline to the 1Y FU** | ***0.001*** | ***0.002*** | | ***0.000*** | |  |
| **BFMDRS-M variation from baseline to the last FU** | ***0.000*** | ***0.000*** | | ***0.000*** | |  |
| **BFMDRS-D variation from baseline to the 1Y FU** | ***0.016*** | ***0.008*** | | ***0.000*** | |  |
| **BFMDRS-D variation from baseline to the last FU** | ***0.003*** | ***0.001*** | | ***0.000*** | |  |
| **TEED variation from baseline to the 1Y FU** | ***0.024*** | *0.102* | | ***0.005*** | |  |
| **TEED variation from baseline to the last FU** | *0.117* | *0.051* | | ***0.015*** | |  |
| **ETIOLOGY: GENETIC/** **HETERODEGENARATIVE** | | | | | |  |
| **BFMDRS-M variation from baseline to the 1Y FU** | ***0.062*** | ***0.008*** | | ***0.000*** | |  |
| **BFMDRS-M variation from baseline to the last FU** | ***0.016*** | ***0.001*** | | ***0.000*** | |  |
| **BFMDRS-D variation from baseline to the 1Y FU** | *0.500* | *0.062* | | ***0.016*** | |  |
| **BFMDRS-D variation from baseline to the last FU** | *0.068* | ***0.002*** | | ***0.001*** | |  |
| **TEED variation from baseline to the 1Y FU** | ***0.031*** | *0.250* | | ***0.011*** | |  |
| **TEED variation from baseline to the last FU** | ***0.016*** | ***0.007*** | | ***0.000*** | |  |
| **ETIOLOGY: IDIOPATHIC** | | | | | |  |
| **BFMDRS-M variation from baseline to the 1Y FU** | ***0.031*** | ***0.014*** | | ***0.000*** | |  |
| **BFMDRS-M variation from baseline to the last FU** | ***0.002*** | ***0.000*** | | ***0.000*** | |  |
| **BFMDRS-D variation from baseline to the 1Y FU** | *0.125* | ***0.006*** | | ***0.000*** | |  |
| **BFMDRS-D variation from baseline to the last FU** | ***0.008*** | ***0.002*** | | ***0.000*** | |  |
| **TEED variation from baseline to the 1Y FU** | *0.125* | ***0.017*** | | ***0.002*** | |  |
| **TEED variation from baseline to the last FU** | *0.695* | *0.127* | | *0.142* | |  |
| **ETIOLOGY: ACQUIRED** | | | | | |  |
| **BFMDRS-M variation from baseline to the 1Y FU** | *0.062* | *0.250* | | ***0.008*** | |  |
| **BFMDRS-M variation from baseline to the last FU** | *0.062* | ***0.031*** | | ***0.001*** | |  |
| **BFMDRS-D variation from baseline to the 1Y FU** | *0.125* | *0.250* | | ***0.016*** | |  |
| **BFMDRS-D variation from baseline to the last FU** | *0.125* | *0.068* | | ***0.011*** | |  |
| **TEED variation from baseline to the 1Y FU** | *0.625* | *0.062* | | *0.074* | |  |
| **TEED variation from baseline to the last FU** | *0.812* | *0.062* | | *0.131* | |  |

**eSupplemental Figure 1.** The BFMDRS-M scores at baseline and follow-ups are presented for all the subjects. Each subject is coded as follows: #progressive registry **number** + **sex** (M or F)_**etiology** [idiop, idiopathic, or genetic/heredodegenerative (with the gene specified) or DCP, dyskinetic/dystonic cerebral palsy)]

**eSupplemental Material S1**

***Optimal stimulation targets analysis***

We defined the optimal stimulation targets in both hemispheres for males and females separately by measuring the weighted centers of the stimulation center points using the following equation:

$$(x_{center}, y_{center}, z_{center})=(\frac{\sum_{i-1}^{n} {improvement}_{i}\times x_{i}}{\sum_{i-1}^{n} {improvement}_{i}},\frac{\sum_{i-1}^{n} {improvement}_{i}\times y_{i}}{\sum_{i-1}^{n} {improvement}_{i}},\frac{\sum_{i-1}^{n} {improvement}_{i}\times z_{i}}{\sum_{i-1}^{n} {improvement}_{i}})$$

We calculated the optimal target points for both hemispheres. We used a two-sample t-test to determine whether the optimal targets for the two sexes were significantly different. The null hypothesis was that the two groups had different optimal targets, and the alternative hypothesis was that they were not equal. We performed these statistical comparisons along the anteroposterior, mediolateral, and dorsoventral axes of the GPi separately. We also examined the linear relationship between improvements in BFMDRS-M scores and the location of stimulation centers in the three anatomical axes of the GPi.

**References**

1. Duga V, Giossi R, Romito LM et al. (2024) Long-Term Globus Pallidus Internus Deep Brain Stimulation in Pediatric Non-Degenerative Dystonia: A Cohort Study and a Meta-Analysis. Mov Disord, 39(7) 1131-1144. https://doi.org/10.1002/mds.29815.
